# TGFBR1*6A and Risk for Colorectal Cancer

**DOI:** 10.34133/cancomm.0033

**Published:** 2026-06-09

**Authors:** Allan M. Johansen, Julie T. Ziegler, Kojo Agyemang, Michael J. Pennison, Hugo Jimenez, Loïc Le Marchand, John L. Hopper, Daniel D. Buchanan, Antonio Di Cristofano, Wencheng Li, Greg Dyson, Ann G. Schwartz, Jennifer L. Beebe-Dimmer, Lara Sucheston-Campbell, Asfar S. Azmi, Wael Sakr, Ralph B. D’Agostino, Carl D. Langefeld, Boris C. Pasche

**Affiliations:** ^1^ Department of Cancer Biology, Wake Forest School of Medicine, Winston-Salem, NC, USA.; ^2^ Department of Biostatistics and Data Science, Division of Public Health Sciences, Wake Forest University School of Medicine, Winston-Salem, NC, USA.; ^3^ Department of Oncology, Karmanos Cancer Institute, Wayne State University, Detroit, MI, USA.; ^4^ Epidemiology Program, University of Hawaii Cancer Center, Honolulu, HI, USA.; ^5^ Centre for Epidemiology and Biostatistics, Melbourne School of Population and Global Health, The University of Melbourne, Melbourne, Victoria, Australia.; ^6^ Colorectal Oncogenomics Group, Department of Clinical Pathology, The University of Melbourne, Parkville, Victoria, Australia.; ^7^ Department of Pathology, University of Melbourne Centre for Cancer Research, Victorian Comprehensive Cancer Centre, Parkville, Victoria, Australia.; ^8^ Department of Developmental and Molecular Biology, Albert Einstein College of Medicine, Bronx, NY, USA.; ^9^ Department of Pathology, Wake Forest University Baptist Medical Center, Winston-Salem, NC, USA.; ^10^ Department of Pathology, Wayne State University, Detroit, MI, USA.; ^11^ Atrium Health Wake Forest Baptist Comprehensive Cancer Center, Wake Forest University School of Medicine, Winston-Salem, NC, USA.; ^12^ Center for Precision Medicine, Wake Forest University School of Medicine, Winston-Salem, NC, USA.

It is estimated that 20% of 30% of colorectal cancer (CRC) cases are inherited, although the genetic drivers of risk are not well established [1]. Transforming growth factor beta (TGF-β) plays a central role in the development and progression of CRC. TGFBR1*6A (6A) is a common polymorphism of *TGFBR1*, an in-frame deletion of 3 GCG repeats coding for Alanine (Ala) within the *TGFBR1* signal sequence. Wild-type *TGFBR1* encodes for 9 Ala (9A) [2]. 6A (rs11466445) is not included in genome-wide association study (GWAS) chips and is commonly missed by next-generation sequencing platforms because of its high content of guanine (G) and cytosine (C) bases, which hamper polymerase chain reaction amplification by formation of secondary structures and the requirement for high melting temperatures. The association of 6A with cancer risk has been investigated in multiple types of cancer, and 6A is part of a haplotype putatively associated with colon cancer risk in humans [3]. The shortcoming of these studies has been the lack of adjustment for genetic ancestry and the investigation of a relatively low number of cases and controls. Here, we report the impact of 6A as a modifier of CRC using a novel knock-in mouse model and extend those findings to humans via a large family-based, case–control study, adjusting for genetic ancestry.

First, we developed a knock-in mouse model in which the wild-type *Tgfbr1* exon 1 is replaced with human 9A or 6A. These mice were crossed with Adenomatous polyposis coli^multiple intestinal neoplasia/+^ (*Apc*^Min**/+**^) mice (Supplementary Materials and Methods). After 12 weeks, intestinal tissue was collected from these mice, and polyp growth was counted and classified by morphology. All mice harboring the 6A allele exhibited significantly decreased polyp formation (*P* = 0.020; Fig. 1A). Specifically, the heterozygous 9A/6A genotype had 49.7% fewer polyps (21.8 ± 2.2) compared with the 9A/9A genotype (43.4 ± 4.1; *P* = 0.014). Although not statistically significant, the number of polyps (28.1 ± 5.4) of 6A/6A mice was also 35.3% lower compared with the 9A/9A mice (*P* = 0.056; Fig. 1A). Subset analysis of small intestinal polyps confirmed these findings. Heterozygous (9A/6A) mice had an average of 19.5 ± 2.0 polyps and homozygous 6A mice had an average of 26.3 ± 5.2 polyps, which were lower (53.1% and 36.6%, respectively) than the homozygous 9A mice (41.6 ± 4.3 polyps, *P* = 0.011 and *P* = 0.050, respectively; Fig. 1B). Analyzing the data under a dominant genetic model for 6A, mice with the 6A allele (9A/6A or 6A/6A genotypes) had 46% fewer total polyps (*P* = 0.020; Fig. 1A) and 48.2% fewer small intestinal polyps (*P* = 0.017; Fig. 1B) compared with 9A/9A mice.

**Fig. 1.**
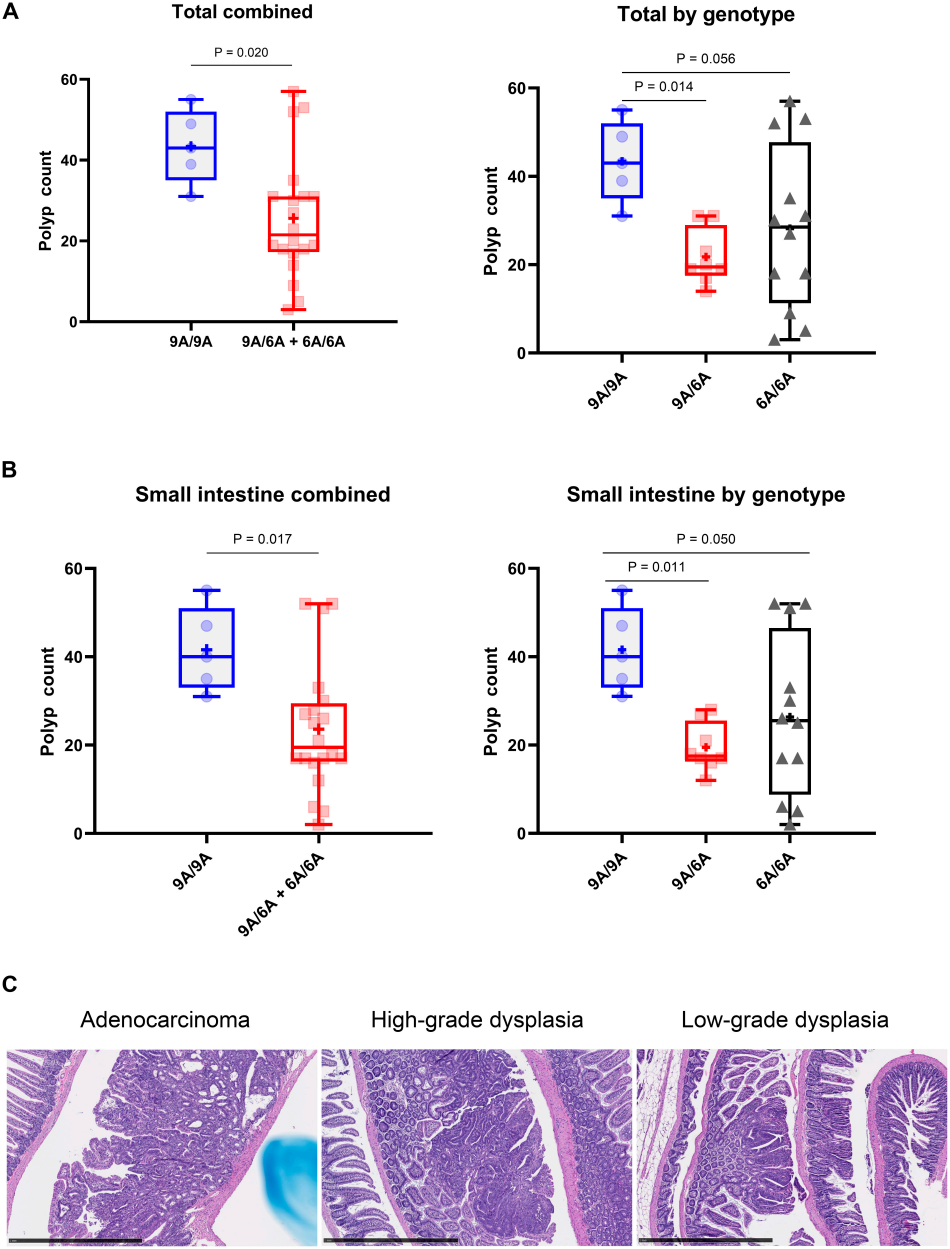
TGFBR1*6A (6A) decreases polyp formation and adenoma to carcinoma transformation in *Apc*^Min/+^ mice. (A) Polyp formation of *Apc*^Min/+^ mice crossed with knock-in TGFBR1*9A (9A) and/or 6A allele, in C57BL/6 mice. Mice were euthanized at 12 weeks, intestinal tissue was collected, and polyps were counted (9A/9A, *n* = 5; 9A/6A, *n* = 8; 6A/6A *n* = 12). Data points indicate the number of tumors in each individual mouse. For combined small intestine and colon tumor count, the 6A carrying variant was significantly lower. The heterozygote 9A/6A compared to the common 9A/9A had a significantly lower total polyp count, while the rare 6A/6A was trending toward significance. (B) Small intestine combined count was significantly lower in mice carrying the 6A variant, and both the 9A/6A heterozygote and the rare 6A/6A had significantly lower polyp count in small intestine. (C) Small intestine tissue representative images (H&E) of the left red circle representing adenocarcinoma, center red circle representing high-grade dysplasia, and right-most circle representing low-grade dysplasia collected from the humanized knock-in mice (scale bar = 1,000 μm). Polyps from 6A/6A (*n* = 6) and 9A/6A (*n* = 5) showed low-grade dysplasia, with one instance of high-grade dysplasia, while 9A/9A (*n* = 3) had either early-stage carcinoma or high-grade dysplasia. *Apc*^^Min/+^^, Adenomatous polyposis coli^multiple intestinal neoplasia^; TGFBR1, Transforming Growth Factor Beta receptor 1.

We observed that both the average small intestine polyp length of 9A/6A (1.74 ± 0.36 mm) and 6A/6A (1.68 ± 0.19 mm) were smaller than that in 9A homozygous mice (3.00 ± 0.50 mm, *P* = 0.080 and *P* = 0.018, respectively). Thus, the average polyp length of the mice with at least one 6A allele was significantly smaller compared with 9A/9A polyp length (1.71 ± 0.18 mm vs. 3.00 ± 0.50 mm, *P* = 0.011; Table S1). Histologically, polyps from 6A/6A (*n* = 6) and 9A/6A (*n* = 5) mice mainly displayed low-grade dysplasia (80.0% to 83.3%), with one instance of high-grade dysplasia, while 9A/9A (*n* = 3) polyps only displayed high-grade dysplasia or early-stage carcinoma (Fig. 1C and Table S2).

We then tested for an association between 6A genotype and CRC in humans. We used the Colon Cancer Family Registry (CCFR) dataset, which included germline DNA for 3,374 individuals and GWAS data for 3,405 cases and relatives (Supplementary Materials and Methods). When performing statistical analyses adjusting for genetic ancestry (Supplementary Materials and Methods), the 6A allele was found to be associated with a decreased risk for CRC (odds ratio [OR] = 0.85, 95% confidence interval [CI] = 0.73 to 0.98, *P* = 0.022) under an additive genetic model (Table S3). Contextually, the population attributable fraction for non-6A carriers was 12.5%, assuming a 4% lifetime risk of CRC. When restricting the analysis to cases and their siblings (87.6% of controls were siblings), which were genetically identical to the mouse model, we found an even larger effect size (OR = 0.56, 95% CI = 0.34 to 0.92, *P* = 0.023; Table S3). Additionally, we used Generalized Estimating Equations to test for association with CRC risk for the *TGFBR1* region single-nucleotide polymorphisms (SNPs). We investigated if tag SNPs selected in the 6A region had any residual independent association with risk for CRC. None of the SNPs were associated with CRC status after adjusting for the 6A polymorphism (Fig. S1 and Table S4). Hence, the functional variant 6A captures the association across the region and is the driving functional allele of the observed effect. Differences between 6A and 9A signaling have been documented in both normal and cancer cells [2,4–6]. Whether 6A signaling affects epithelial and/or stromal impact on adenoma development will need to be further studied.

Next, we repeated the analyses stratifying or subsetting by key demographic and clinical characteristics of the cases (Tables S5 and S6). Stratifying by age decades, a generally consistent pattern of OR < 1.00 was observed, except for age stratum 60 to 69 (Table S5). We noted that the number of homozygotes for 6A were very small, resulting in limited statistical power for any individual stratum analysis and only the age stratum ≥70 reached statistical significance (OR = 0.49, 95% CI = 0.29 to 0.83, *P* = 0.007; Table S5). Subsetting to individuals positive for some clinical and pathological variables reduced the sample size. Subsetting for familial adenomatous polyposis was statistically significant (OR = 0.16, 95% CI = 0.03 to 0.87, *P* = 0.035) for 27 cases and 10 controls (Table S5).

The data presented in this study showed that 6A was associated with reduced risk of CRC. The concordant mouse and human findings support the idea that 6A is a high-frequency, low-penetrance CRC protective allele. The knock-in mouse model reported here demonstrated that mice carrying 6A developed 46% fewer polyps than 9A/9A mice, had smaller polyps, had lower-grade dysplasia, and did not harbor carcinoma (Fig. 1). Similarly, there was an almost identical 44% decreased CRC risk (OR = 0.56) among siblings carrying the 6A allele (Table S3), a population that closely mimics the humanized mouse model because of its shared genetic background. In patients with familial polyposis, a disease caused by germline mutation of the *APC* gene, 6A may be considered a high-penetrance protective allele as CRC risk was decreased by 84% (OR = 0.16) (Table S5). This strongly suggests that 6A is a potent modifier of *APC*-mediated CRC tumorigenesis. Importantly, the association of decreased CRC risk was also observed among all cases and controls, demonstrating the applicability of these findings to the general population (Table S3). By adjusting the CCFR analyses for potential confounding factors, including genetic ancestry admixture, we attempted to address the limitations of several prior 6A studies, acknowledging that further studies of other ancestral populations and ethnicities are required to generalize the findings to those populations (e.g., Asian and African ancestry, Hispanic ethnicity) [7–9]. Furthermore, these findings add to the evidence that structural variants such as various length insertion–deletions (indels) are also important drivers of risk in diseases. Generally, structural variants are understudied due to challenges in variant calling, and accurate identification and genotyping of short and medium indels remains a challenge, especially for guanine–cytosine (GC)-rich regions, as sequencing has mainly focused on one base pair SNP changes of fixed length [10]. The results presented in this report provide compelling evidence that GWAS-based case–control studies may have overlooked some functional hard-to-genotype GC-rich short indels.

In conclusion, we showed that 6A is a protective risk allele, which markedly reduces CRC risk in discordant sibling pairs. CRC risk reduction is highest in patients with familial adenomatous polyposis. If these findings can be replicated and validated, they could have impactful clinical implications, given the significantly reduced risk of CRC in familial adenomatous polyposis patients and among siblings of patients carrying the 6A gene.

## Ethical Approval

All subjects signed an informed consent form prior to giving their information to the CCFR. The institutional review board (IRB) for each CCFR site participated in ethics approval and was approved by the Wake Forest University IRB (IRB00029552) committee for research use. The animal study was reviewed and approved by the University of Alabama at Birmingham Institutional Animal Care and Use Committee (animal project number: 140108941).

## Data Availability

All data are available in the main text or the Supplementary Materials. GWAS data can be requested from the CCFR by procedural application.
