# Supplementary material for: TGFBR1*6A and Risk for Colorectal Cancer
Source: Cancer Commun (Lond). 2026 Jun 9;46:0033. doi: 10.34133/cancomm.0033 (PMC13247310; doi:10.34133/cancomm.0033)
Supplement: Supplementary 1 — Figs. S1 and S2 Tables S1 to S8 [file cancomm.0033.f1.docx]

**Supplementary Materials**

***TGFBR1**6A and Risk for Colorectal Cancer**

Allan M. Johansen^1^, Julie T. Ziegler^2^, Kojo Agyemang^1^, Michael J. Pennison^1^, Hugo Jimenez^3^, Loic Le Marchand^4^, John L. Hopper^5^, Daniel D. Buchanan^6,7^, Antonio Di Cristofano^8^, Wencheng Li^9^, Greg Dyson^3^, Ann G. Schwartz^3^, Jennifer L. Beebe-Dimmer^3^, Lara Sucheston-Campbell^3^, Asfar S. Azmi^3^, Wael Sakr^10^, Ralph B. D’Agostino Jr.^2^, Carl D. Langefeld^2,11,12^, Boris C. Pasche^3,*^

^1^Department of Cancer Biology, Wake Forest School of Medicine, Winston-Salem, NC, USA.

^2^Department of Biostatistics and Data Science, Division of Public Health Sciences, Wake Forest University School of Medicine, Winston-Salem, NC, USA.

^3^Department of Oncology, Karmanos Cancer Institute, Wayne State University, Detroit, MI, USA.

^4^Epidemiology Program, University of Hawaii Cancer Center, Honolulu, HI, USA.

^5^Centre for Epidemiology and Biostatistics, Melbourne School of Population and Global Health, The University of Melbourne, Melbourne, Victoria, Australia.

^6^Colorectal Oncogenomics Group, Department of Clinical Pathology, The University of Melbourne, Parkville, Victoria, Australia.

^7^ Department of Pathology, University of Melbourne Centre for Cancer Research, Victorian Comprehensive Cancer Centre, Parkville, Victoria, Australia.

^8^ Department of Developmental and Molecular Biology Albert Einstein College of Medicine, Bronx, NY, USA.

^9^Department of Pathology, Wake Forest University Baptist Medical Center, Winston-Salem, NC, USA.

^10^Department of Pathology, Wayne State University, Detroit, MI, USA.

^11^Atrium Health Wake Forest Baptist Comprehensive Cancer Center, Wake Forest University School of Medicine, Winston-Salem, NC, USA.

^12^Center for Precision Medicine, Wake Forest University School of Medicine, Winston-Salem, NC, USA.

^*^Corresponding author: Boris C. Pasche, pascheb@karmanos.org.

**Supplementary Materials and Methods**

**Mouse model**

*TGFBR1**6A (6A) is only found in humans and non-human primates. Mice harbor a three GCG repeat sequence within the *Tgfbr1* signal sequence. We therefore designed a novel knock-in mouse model carrying humanized *TGFBR1**9A (9A) or 6A by replacing the mouse *Tgfbr1* exon 1 with human *TGFBR1* exon 1.

A targeting vector was designed by Ozgene (Bentley, Australia) using proprietary backbone and selection cassettes to remove the mouse *Tgfbr1* exon 1 starting from the ATG start codon, which was then replaced with human *TGFBR1* exon 1 carrying either 9A or 6A.

Following homologous recombination of the targeting vector in 129SvIm mouse embryonic stem cells (Stratagene, CA, USA), the knock-in 129SvIm embryonic stem cells, identified by PCR were, grown in Dulbecco’s modified Eagle’s medium (DMEM) supplemented with 15% fetal calf serum + 1 mM glutamine + 0.1 mM β-mercaptoethanol + 1% nonessential amino acids + Leukemia Inhibitory Factor (LIF, Sigma, MA, USA), on mitotically inactivated mouse embryonic fibroblasts. They were karyotyped and injected into C57BL/6 mouse blastocysts (The Jackson Laboratory, ME, USA) to establish germline transmission and a colony.

Mice, acquired from the Jackson Laboratory, were housed in a specific pathogen-free facility under a 12 h light/12 h dark cycle at controlled temperature and humidity, with ad libitum access to standard chow and water. Animals were group-housed (up to 5 mice per cage) in ventilated cages containing standard bedding and environmental enrichment. Cage changes and routine husbandry were performed according to institutional animal care guidelines.

Fully congenic (99.9%) C57BL/6 background mice were bred by backcrossing the F3 129SvIm C57BL/6 mice with C57BL/6 mice and then confirmed using speed congenics genome-wide panel of 150 SNP markers (The Jackson Laboratory, ME, USA). The mice were bred and genotyped to confirm 9A or 6A knock-in, using 5’-CGAGGCTTCCTGAGGAGAAG-3’ and 5’-CGCATTTAGGCAGTGGAATC-3’ primers (IDTDNA, IA, USA). Knock-in mice were mated with C57BL/6J *Apc*^Min/+^ mice to generate mice with human *TGFBR1* exon 1, either with or without the three-alanine deletion (6A or 9A respectively). The *Apc*^Min/+^ locus was detected as previously described [1].

**Mouse intestinal tissue collection and staining**

At 12 weeks, mice were euthanized by CO2 narcosis followed by cervical dislocation. Humane endpoints were determined following IACUC guidelines based on clinical scoring criteria (body weight, body condition, activity, and appearance). No mice with signs of distress or unrelated health concerns were euthanized before 12 weeks. Following euthanasia, the intestinal tract was collected and divided into the small intestine and colon at the caecum. Intestinal lumen was cleared with sterile PBS and tissue was cut longitudinally for gross evaluation and fixed in 10% neutral buffered formalin (e.g., 10% Neutral Buffered Formalin, Fisher Scientific) before routine processing and paraffin-embedding (Lonza Bioscience). Polyps were counted and classified by morphology, using a Leica S6D Dissecting Microscope with a Schott ACE I light source (Leica Microsystems), by two independent researchers, as previously described [1].

After fixation and embedding, hematoxylin and eosin (H&E) slides were prepared at Wake Forest Baptist Hospital Pathology Department for further analysis. Briefly, paraffin‑embedded sections were deparaffinized in xylene, rehydrated through graded ethanol, and stained with hematoxylin to visualize nuclei. After differentiation and bluing, slides were counterstained with eosin to highlight cytoplasmic and extracellular components, followed by dehydration, clearing, and coverslipping for microscopic evaluation. All images were prepared with Nano Zoomer Digital Pathology software (Agilent Technologies, CA, USA) from digital slide scans (2.5× magnification). An independent, blinded pathologist performed tissue analysis.

A two-tiered stratification system (low-grade and high-grade dysplasia) was used to grade the severity of dysplasia. Low-grade dysplasia was defined by the presence of enlarged, hyperchromatic nuclei, with varying degrees of nuclear spindling and stratification. High-grade dysplasia was characterized by marked complex glandular crowding and irregularity of glands, cribriform architecture, and intraluminal necrosis as architectural features. The size of the polyps was measured at their longest dimension using the Nano Zoomer Digital Pathology software (Hamamatsu Photonics K.K., Hamamatsu City, Japan).

**Study participants**

The Colon Cancer Family Registry (CCFR) is an international consortium of six institutions in North America and Australia-Asia [2, 3]. The consortium completed multi-site recruitment from 1997 to 2012, and collected clinical data, pathological samples, and epidemiological data via questionnaires [2]. The CCFR used two recruitment strategies: population-based (case probands from cancer registries) and clinic-based (families with multiple cases of CRC) [2]. We acquired germline DNA from 3,374 participating family members from the CCFR and 3,405 accompanying epidemiological data [2]. CCFR recruited cases and controls from a survival cohort and an unaffected cohort of relatives [2]. Men and women with CRC (cases), first- and second-degree relatives without CRC (controls) granted the CCFR access to their medical records and were followed up every five years to update their cancer screening and family history. All subjects signed an informed consent form prior to giving their information to the CCFR. The institutional review board (IRB) for each CCFR site participated in ethics approval and was approved by the Wake Forest University IRB committee for research use.

**CCFR**

The CCFR data set included germline DNA for 3,374 individuals and GWAS data for 3,405 cases and relatives, representing all CCFR centers (centers 11-16). The CCFR participants were genotyped in four groups using four arrays: CCFR Set-1 (Illumina 1M/1M-Duo) and Set-2 (Illumina Omni1-Quad), CCFR Set-3 (Affymetrix Axiom CORECT Set array), and CCFR Set-4 (Illumina OncoArray 600K SNP array) [4-7]. Initial genotype quality control was completed by the CCFR Central Informatics Centre, University of Melbourne. In addition, we computed standard quality control analyses for genotype quality (missingness rates for individuals, missingness rates for single nucleotide polymorphisms (SNPs), relatedness, departures from Hardy-Weinberg Proportions Expectations). Population substructure and ancestry estimates were computed using the software ADMIXTURE (Admixture studio, CA, USA), with HapMap CEU, YRI, CHB, and MEX samples as reference anchoring populations. Given the small number of individuals not of European ancestry, the analyses were restricted to self-reported and genetically consistent European Americans, as characterized by the anchoring HapMap populations (Supplementary Figure S2A).

**Genotyping**

Whole-genome amplification was performed on germline DNA for 3,374 individuals. After exclusion of poor DNA quality samples, at PicoGreen >50 ng/µL threshold, we genotyped 1,638 controls and 772 cases from the CCFR centers 1-5, total of 2,410 participants (Supplementary Table S7). Primers were purchased from IDT-DNA (IDTDNA, IA, USA) with forward sequence 5’-GAGGCGAGGTTTGCTGGGGTGAGGCA-3’ and reverse sequence 5’-CATGTTTGAGAAAGAGCAGGAGCGAG-3’. Amplification was done using Platinum *Tsp* polymerase (Thermofischer, MA, USA) for 30× cycles, using manufacturers protocol with the addition of 2M Betaine (Sigma Aldrich, MI, USA) for GC-rich regions. The amplicon double restriction digested with XmaI and BssSaI (New England Biolabs, MA, USA) and resolved on 10% Criterion™ TBE-Urea Polyacrylamide (BioRad, CA, USA). For optimal separation, the electrophoresis-chambers were run in ice baths. Samples were stained with GelRed® Nucleic Acid Gel Stain (Sigma Aldrich, MI, USA) and imaged with a myECL imaging device (Thermo Fisher Scientific, MA, USA). Genotyping interpretation was done by committee review, which consisted of two additional double-blinded investigators in addition to the primary reviewer.

**Statistical analyses**

Murine study: genotype data were tested for departures from Hardy-Weinberg Equilibrium using a chi-square goodness of fit test. A chi square test of independence and Fisher’s exact test was computed to test for an association between mouse polyp counts and genotypes 6A/9A genotypes; both genotypic and allelic test were computed. The polyp count and dimensions were compared by a one-way analysis of variance (ANOVA).

CCFR study: initial genotype quality control was completed by the CCFR Central Informatics Centre, University of Melbourne.

In 3,405 samples from the CCFR with GWAS data, we imputed the *TGFBR1* region of chromosome 9 using the 1,000 Genomes phase 3 reference panel (Supplementary Figure S2B, Supplementary Table S8). Specifically, the program ShapeIt (https://mathgen.stats.ox.ac.uk/genetics_software/shapeit/shapeit.html) was used to pre-phase the data for SNPs that passed quality control and were within 1.4 Mb of rs11466445. Impute2 (University of Oxford, United Kingdom) was used to impute the genotypes for the rs11466445 SNP [8]. To verify imputation accuracy, we compared the imputed “best guess” genotypes with the 999 existing genotyped samples from the CCFR that were directly genotyped for rs11466445 with the GWAS data (97.3% genotype-imputation concordance rate) (Supplementary Figure S2B). Given the concordance rate, best guess genotypes were used throughout. After our exclusion criteria we had 3,299 6A imputed genetically determined samples of European Ancestry genotypes.

To test the hypothesis that the 6A polymorphism is associated with CRC, we used two parallel analytic approaches to account for family structure. First, we computed a generalized estimating equations (GEE1) using the logit link and the robust (sandwich) estimator. We included sex and three population substructure proportions as covariates. We also, as a robustness check, computed a generalized linear mixed model (logit link) with family as a random effect and sex and three population substructure proportions were included as fixed effects.

To test for residual variance, we used previously identified tag SNPs reported in Pasche et al. [9], that captured the genetic variation across the *TGFBR1* region proximal to the 6A locus. The study selected 6A and 18 additional haplotype tag SNPs, using the phase II HapMap data and the CEU population, to cover pairwise r^2^ > 0.8 for all common SNPs in the *TGFBR1* region, including 6A [9]. We investigated if tag SNPs selected to capture the percent variation in the region containing the 6A polymorphism, had any residual independent association with CRC risk (Supplementary Figure S1).

We assumed a CRC incidence rate of approximately 1 in 25 (~4%) to compute the population attributable fraction (PAF) for incident cases [10]. For details, please refer to Siegel et al. [10]. Following the equation described in the literature, PAF was computed using the R package graphPAF (R studio, New Zealand) [11].

**Supplementary Table S1. Polyp length of small intestines in *Apc*^min/+^ *TGFBR1**6A (6A) and *TGFBR1**9A (9A) mice.**

| **Genotype** | **Average polyp length**  **(mm)** | **SEM** | ***P* value**  **(referenced to 9A/9A)** |
| --- | --- | --- | --- |
| 9A/9A | 3.00 | 0.50 | N/A |
| 9A/6A | 1.74 | 0.36 | 0.080 |
| 6A/6A | 1.68 | 0.19 | 0.018 |
| 9A/6A + 6A/6A | 1.71 | 0.18 | 0.011 |

Abbreviations: *Apc*^min/+^, Adenomatous Polyposis Colimultiple^intestinal neoplasia/+^; 9A, *TGFBR1* wild-type carries of 9 Alanine’s; 6A, *TGFBR1* deletion carries of 6 Alanine’s; SEM, standard error of mean; N/A, not applicable.

**Supplementary Table S2. Small intestine polyp morphology of *Apc*^min/+^ *TGFBR1**6A (6A) and *TGFBR1**9A (9A) mice.**

| **Genotype** | **Early-stage carcinoma,**  ***n* (%)** | **High-grade dysplasia,**  ***n* (%)** | **Low-grade dysplasia,**  ***n* (%)** |
| --- | --- | --- | --- |
| 9A/9A | 2 (66.7) | 1 (33.3) | 0 (0) |
| 9A/6A | 0 (0) | 1 (20.0) | 4 (80.0) |
| 6A/6A | 0 (0) | 1 (16.7) | 5 (83.3) |

Abbreviations: *Apc*^min/+^, Adenomatous Polyposis Colimultiple^intestinal neoplasia/+^; 9A, *TGFBR1* wild-type carries 9 Alanines; 6A, *TGFBR1* deletion carries 6 Alanines; SEM, standard error of mean.

**Supplementary Table S3. Primary CRC association results for *TGFBR1**6A (6A) in CCFR^a^.**

| **Characteristics** | **Genetic model** | **β** | **SE(β)** | ***P* value** | **OR** | **95%CI** | **9A/9A** | | **9A/6A** | | **6A/6A** | |
| --- | --- | --- | --- | --- | --- | --- | --- | --- | --- | --- | --- | --- |
|  |  |  |  |  |  |  | **Case** | **Control** | **Case** | **Control** | **Case** | **Control** |
| Overall | add | -0.167 | 0.073 | 0.022 | 0.85 | 0.73-0.98 | 1,720 | 930 | 374 | 235 | 21 | 19 |
| Siblings | add | -0.584 | 0.256 | 0.023 | 0.56 | 0.34-0.92 | 103 | 815 | 16 | 203 | 0 | 17 |

^a^Overall CCFR GWAS-based case-control cancer risk association results and subset to sibling controls only. A generalized estimating equations (GEE1) using a logit link and adjusting for genetic ancestry and genetically verified sex was computed to test for association assuming an additive model. To account for familial correlation, the sandwich estimator of the variance was computed. Major allele = 9A, minor allele = 6A.

Abbreviations: add, additive genetic model; SE, Standard Error OR, Odds Ratio; CI, Confidence Interval; CCFR, Colon Cancer Family Registry.

**Supplementary Table S4. Estimated linkage disequilibrium across linkage disequilibrium blocks in the *TGFBR1**6A (6A) region in the CCFR population^a^.**

| **SNP** | **β** | **SE(β)** | ***P* value** | **OR** | **95%CI** |
| --- | --- | --- | --- | --- | --- |
| **rs7034462** | -0.0049 | 0.1699 | 0.977 | 1.00 | 0.70-1.38 |
| **rs1888223** | -0.0002 | 0.0574 | 0.997 | 1.00 | 0.89-1.11 |
| **rs6478974** | -0.0668 | 0.0491 | 0.174 | 0.94 | 0.79-0.96 |
| **rs10739778** | 0.0168 | 0.0538 | 0.756 | 1.02 | 0.93-1.14 |
| **rs2026811** | 0.0144 | 0.0560 | 0.797 | 1.01 | 0.92-1.14 |
| **rs11568785** | -0.0278 | 0.2118 | 0.896 | 0.97 | 0.62-1.43 |
| **rs10733710** | 0.0854 | 0.0577 | 0.139 | 1.09 | 1.05-1.32 |
| **rs334349** | -0.0247 | 0.0530 | 0.641 | 0.98 | 0.85-1.05 |
| **rs1626340** | -0.0447 | 0.0564 | 0.428 | 0.96 | 0.81-1.02 |

^a^Estimated linkage disequilibrium across linkage disequilibrium blocks in the 6A region in the CCFR population. Colon cancer risk association results for 6A adjusted for clinical and pathological covariates. Data was analyzed using GEE1 with sandwich estimator while adjusting for genetic ancestry, sex and specific covariate data points. Major allele = 9A, minor allele = 6A.

Abbreviations: TGFBR1, Transforming Growth Factor Beta Receptor 1; GEE1, generalized estimating equations using the sandwich estimator of the variance; CCFR, Colon Cancer Family Registry; SNP, Single Nucleotide Polymorphism; SE, Standard Error; OR, Odds Ratio; CI, Confidence Interval.

**Supplementary Table S5. CRC risk association results for *TGFBR1**6A (6A) stratified by clinical and pathological covariates in CCFR GWAS participants****^a^.**

| **Characteristics** | **Genetic model** | **β** | **SE(β)** | ***P* value** | **OR** | **95%CI** | **9A/9A** | | **9A/6A** | | **6A/6A** | |
| --- | --- | --- | --- | --- | --- | --- | --- | --- | --- | --- | --- | --- |
|  |  |  |  |  |  |  | **Case** | **Control** | **Case** | **Control** | **Case** | **Control** |
| **Age, years** | | | | | | | | | | | | |
| Under 40 | dom | -0.073 | 0.257 | 0.776 | 0.93 | 0.56-1.54 | 133 | 96 | 32 | 26 | 2 | 1 |
| 40-49 | add | -0.163 | 0.144 | 0.259 | 0.85 | 0.64-1.13 | 566 | 288 | 105 | 61 | 7 | 6 |
| 50-59 | add | -0.152 | 0.128 | 0.235 | 0.86 | 0.67-1.10 | 512 | 303 | 125 | 85 | 9 | 9 |
| 60-69 | dom | 0.119 | 0.223 | 0.593 | 1.13 | 0.73-1.74 | 321 | 168 | 72 | 31 | 2 | 2 |
| 70 and over | dom | -0.714 | 0.266 | 0.007 | 0.49 | 0.29-0.83 | 188 | 75 | 40 | 32 | 1 | 1 |
| **Crohns** | dom | 0.635 | 1.041 | 0.542 | 1.89 | 0.25-14.5 | 15 | 3 | 11 | 1 | 0 | 0 |
| **Familial adenomatous polyposis** | dom | -1.849 | 0.875 | 0.035 | 0.16 | 0.03-0.87 | 22 | 7 | 5 | 3 | 0 | 0 |
| **Smoker** | dom | 0.068 | 0.531 | 0.899 | 1.07 | 0.38-3.03 | 79 | 40 | 13 | 6 | 0 | 0 |
| **Alcohol consumption age** | | | | | | | | | | | | |
| At age 20 | add | -0.104 | 0.095 | 0.273 | 0.90 | 0.75-1.09 | 1,107 | 616 | 244 | 145 | 15 | 13 |
| At age 30 | add | -0.110 | 0.098 | 0.263 | 0.90 | 0.74-1.09 | 1,045 | 578 | 220 | 136 | 17 | 12 |
| At age 50 | dom | -0.191 | 0.160 | 0.234 | 0.83 | 0.60-1.13 | 511 | 284 | 114 | 75 | 4 | 4 |

^a^CRC risk association results for 6A stratified by clinical and pathological covariates in CCFR GWAS samples. A generalized estimating equations (GEE1) using the sandwich estimator of the variance and assuming a logit link and additive genetic model while adjusting for genetic ancestry and genetically verified sex was computed to test for association. If there was a lack of fit to the additive model, the minimum of the additive, dominant, and recessive p-values is reported, with the restriction that N_6A/6A_ ≥ 30 for a recessive model and N_6A/6A_ ≥ 10 for an additive model.

Abbreviations: dom, dominant genetic model; add, additive genetic model; *TGFBR1*, Transforming Growth Factor Beta Receptor 1; CCFR, Colon Cancer Family Registry; GWAS, Genome Wide Association Study; SE, Standard Error; OR, Odds Ratio; CI, Confidence Interval.

**Supplementary Table S6.** **Clinical and pathological features of CRC cases from the CCFR participants.**

| **Characteristics** | **Case**  **(*n* = 2,115)** | **Control**  **(*n* = 1,184)** |
| --- | --- | --- |
| **Disease** | | |
| Diabetes, *n* (%) | 162 (7.69) | 73 (6.20) |
| Crohn’s, *n* (%) | 26 (1.25) | 4 (0.34) |
| Colitis, *n* (%) | 72 (3.45) | 14 (1.20) |
| Familial adenomatous polyposis, *n* (%) | 27 (1.33) | 10 (0.86) |
| Polyps, *n* (%) | 1,070 (51.8) | 305 (26.1) |
| Adenomatous polyps, *n* (%) | 215 (26.6) | 64 (25.0) |
| **Medication** | | |
| Aspirin, *n* (%) | 623 (29.8) | 337 (29.1) |
| Ibuprofen, *n* (%) | 409 (19.6) | 280 (24.0) |
| COX2 inhibitor, *n* (%) | 5 (6.17) | 2 (3.45) |
| **Life style** | | |
| Weight in kg 2 years ago, median (range) | 79 (36-160) | 77 (43-160) |
| Weight in kg at age 20, median (range) | 65 (33-160) | 63 (33-159) |
| Alcohol use once per week in 20s, *n* (%) | 1,366 (65.7) | 774 (65.7) |
| Alcohol use once per week in 30s and 40s, *n* (%) | 1,282 (62.4) | 726 (63.6) |
| Alcohol use once per week over 50, *n* (%) | 629 (53.1) | 363 (54.1) |
| Ever cigarette smoker, *n* (%) | 92 (20.5) | 46 (20.5) |
| Ever cigar or pipe smoker, *n* (%) | 198 (11.5) | 107 (10.9) |

Abbreviations: CCFR, Colon Cancer Family Registry; COX2, Cyclooxygenase 2.

**Supplementary Table S7. Demographics for directly genotyped cases and close relatives.**

| **Characteristics** | **Case**  **(*n* = 772)** | **Control**  **(*n* = 1,638)** |
| --- | --- | --- |
| **Sex** | | |
| Male, *n* (%) | 397 (51.4) | 711 (43.4) |
| Female, *n* (%) | 375 (48.6) | 927 (56.6) |
| **Race** | | |
| White, *n* (%) | 494 (64) | 1,133 (69.2) |
| Black, *n* (%) | 10 (1.3) | 12 (0.7) |
| Hispanic, *n* (%) | 13 (1.7) | 29 (1.8) |
| Asian, *n* (%) | 74 (9.6) | 148 (9) |
| Native American, *n* (%) | 13 (1.7) | 15 (0.9) |
| Unknown, *n* (%) | 168 (21.8) | 301 (18.4) |
| ***TGFBR1* genotype** | | |
| 9A/9A, *n* (%) | 693 (89.8) | 1,417 (86.5) |
| 9A/6A, *n* (%) | 71 (9.2) | 214 (13.1) |
| 6A/6A, *n* (%) | 8 (1) | 7 (0.4) |
|  |  |  |

Abbreviations: TGFBR1, Transforming Growth Factor Beta Receptor 1.

**Supplementary Table S8.** **Demographics of the CCFR GWAS participants.**

| **Characteristics** | **Case**  **(*n* = 2,115)** | **Control**  **(*n* = 1,184)** |
| --- | --- | --- |
| **Age of participants, years, median (range)** | 52 (23-90) | 52 (22-90) |
| **Age of participants, years, mean ± SD** | 53.7 ± 11.0 | 53.1 ± 11.4 |
| **Sex, *n* (%)** |  |  |
| Male | 1,078 (51.0) | 530 (44.8) |
| Female | 1,037 (49.0) | 654 (55.2) |
| **Age, years; *n* (%)** | | |
| Under 40 | 167 (7.9) | 123 (10.4) |
| 40-49 | 678 (32.1) | 355 (30.0) |
| 50-59 | 646 (30.5) | 397 (33.5) |
| 60-69 | 395 (18.7) | 201 (17.0) |
| 70 and over | 229 (10.8) | 108 (9.1) |
| **Recruitment type, *n* (%)** | | |
| Population | 1,806 (85.4) | 1,155 (97.6) |
| Clinic | 309 (14.6) | 29 (2.5) |

Abbreviations: SD, Standard deviation; CCFR, Colon Cancer Family Registry; GWAS, Genome Wide Association Study.


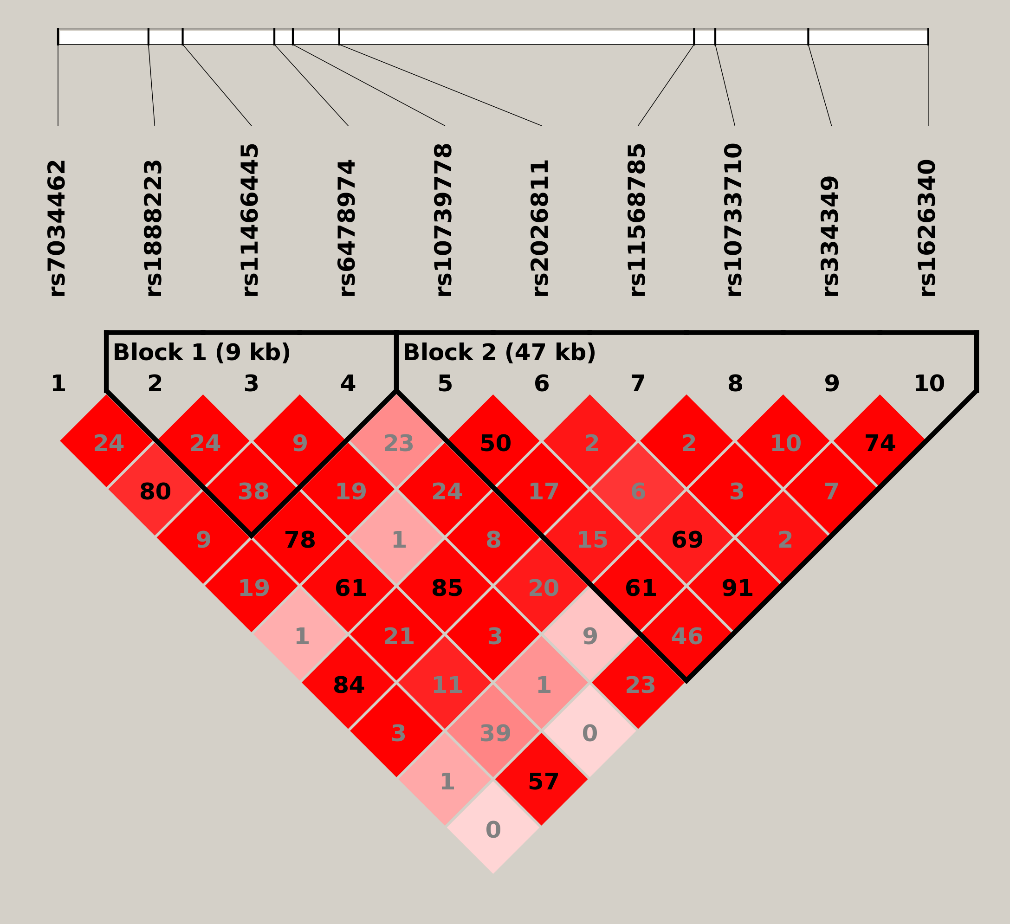


**Supplementary Figure S1. LD hap map of *TGFBR1**6A region.**

Analysis of tag SNPs selected to capture the percent variation of the region with *TGFBR1**6A (6A), for evidence of association adjusting for sex and three ancestral proportions anchored by four HapMap populations (CHB, CEU, MEX, and YRI). GEE1 analyses of each tag SNP, adjusted for 6A in CRC for CCFR individuals of European ancestry. No additional SNPs met the significance threshold of 0.1 for entry into this model. All SNPs were analyzed under an additive genetic model assumption. The cell color intensity reflects a combination of D' and LOD score (log₁₀ of the likelihood odds ratio for LD versus no LD), with lighter colors indicating lower D' and/or lower LOD values. The numbers within the cells represent the pairwise LD metric, *r²*.

Abbreviations: LD, Linkage-Disequilibrium; SNP, Single Nucleotide Polymorphism; TGFBR1, Transforming Growth Factor Beta Receptor 1; CHB, Han Chinese in Beijing (China); CEU, Utah residents with Northern and Western European ancestry from the CEPH collection; MEX, Mexican ancestry in Los Angeles (US); YRI, Yoruba in Ibadan (Nigeria); GEE1, Generalized estimating equations using the sandwich estimator of the variance.

**A**

**
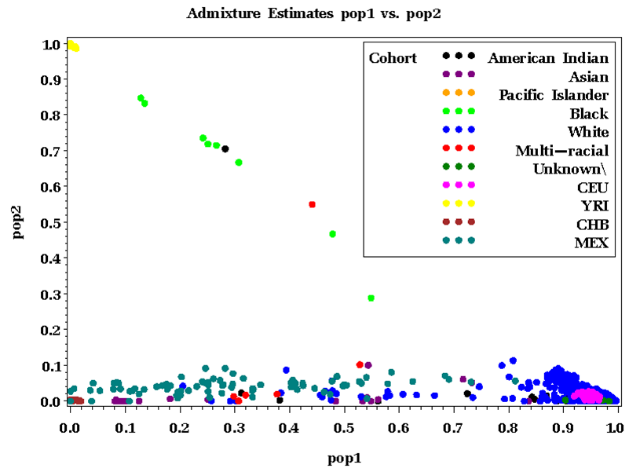
**

**B**


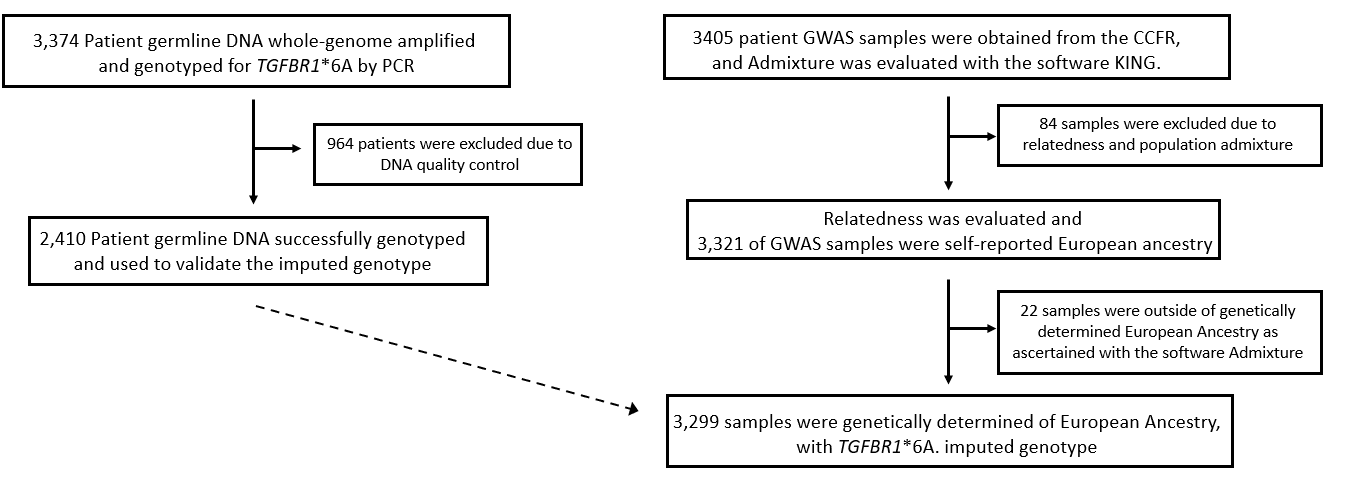


**Supplementary Figure S2. Genotyping and genetic ancestral variance in GWAS samples.**

(A) Admixture analysis was computed to estimate ancestral proportions using the GWAS samples and the HapMap CEU, YRI, CHB, and MEX samples as anchoring populations, using ADMIXTURE. For association analysis, the dataset was restricted to self-reported European Americans with admixture estimate 1 > 0.76 and admixture estimate 2 < 0.20 (*n* = 3,299). Each circle represents an individual sample, with distinct colors indicating the GWAS cohort and the respective HapMap reference populations.

(B) Flow-chart depicting process of case-control acquisition from the CCFR, and inclusion and exclusion criteria for cases and controls genotyped for 6A. The GWAS data on chromosome 9 within 1.4 Mb of the *TGFBR1* region was used to impute the 6A variant.

Abbreviations: GWAS, Genome Wide Association Study; CHB, Han Chinese in Beijing (China); CEU, Utah residents with Northern and Western European ancestry from the CEPH collection; MEX, Mexican ancestry in Los Angeles (US); YRI, Yoruba in Ibadan (Nigeria); CCFR, Colon Cancer Family Registry; Mb, Mega base (Million base pairs).

**Supplementary References**

1. Zeng Q, Phukan S, Xu Y, Sadim M, Rosman DS, Pennison M, et al. Tgfbr1 haploinsufficiency is a potent modifier of colorectal cancer development. Cancer Res. 2009;69(2):678-86.

2. Newcomb PA, Baron J, Cotterchio M, Gallinger S, Grove J, Haile R, et al. Colon Cancer Family Registry: an international resource for studies of the genetic epidemiology of colon cancer. Cancer Epidemiol Biomarkers Prev. 2007;16(11):2331-43.

3. Jenkins MA, Win AK, Templeton AS, Angelakos MS, Buchanan DD, Cotterchio M, et al. Cohort Profile: The Colon Cancer Family Registry Cohort (CCFRC). Int J Epidemiol. 2018;47(2):387-8i.

4. Bien SA, Su YR, Conti DV, Harrison TA, Qu C, Guo X, et al. Genetic variant predictors of gene expression provide new insight into risk of colorectal cancer. Hum Genet. 2019;138(4):307-26.

5. Figueiredo JC, Lewinger JP, Song C, Campbell PT, Conti DV, Edlund CK, et al. Genotype-environment interactions in microsatellite stable/microsatellite instability-low colorectal cancer: results from a genome-wide association study. Cancer Epidemiol Biomarkers Prev. 2011;20(5):758-66.

6. Schumacher FR, Schmit SL, Jiao S, Edlund CK, Wang H, Zhang B, et al. Genome-wide association study of colorectal cancer identifies six new susceptibility loci. Nat Commun. 2015;6:7138.

7. Schmit SL, Edlund CK, Schumacher FR, Gong J, Harrison TA, Huyghe JR, et al. Novel Common Genetic Susceptibility Loci for Colorectal Cancer. J Natl Cancer Inst. 2019;111(2):146-57.

8. Howie BN, Donnelly P, Marchini J. A flexible and accurate genotype imputation method for the next generation of genome-wide association studies. PLoS Genet. 2009;5(6):e1000529.

9. Pasche B, Wisinski KB, Sadim M, Kaklamani V, Pennison MJ, Zeng Q, et al. Constitutively decreased TGFBR1 allelic expression is a common finding in colorectal cancer and is associated with three TGFBR1 SNPs. J ExpClin Cancer Res. 2010;29:57.

10. Siegel RL, Kratzer TB, Giaquinto AN, Sung H, Jemal A. Cancer statistics, 2025. CA Cancer J Clin. 2025;75(1):10-45.

11. Ferguson J, O'Connell M. Estimating and displaying population attributable fractions using the R package: graphPAF. Eur J Epidemiol. 2024;39(7):715-42.
